# A phase II pilot randomized controlled trial to assess the feasibility of the “supra-marginal” surgical resection of malignant glioma (G-SUMIT: Glioma supra marginal incision trial) study protocol

**DOI:** 10.1186/s40814-022-01104-1

**Published:** 2022-07-05

**Authors:** Alireza Mansouri, Carolyn Lai, Damon Scales, Farhad Pirouzmand

**Affiliations:** 1 Department of Neurosurgery, Penn State University, State College, USA; 2 Sunnybrook Health Sciences Centre, Sunnybrook Research Institute, University of Toronto, Toronto, Canada

**Keywords:** High-grade gliomas, Supra-marginal resection, Malignant brain tumor

## Abstract

**Background:**

High-grade gliomas are the most common primary malignant brain tumor in adults having a median survival of only 13–16 months. This is despite the current standard of maximal safe surgical resection followed by fractionated radiotherapy and chemotherapy. Extending the tumor resection limit beyond the gadolinium (GAD)-enhancing margin (i.e., supra-marginal resection) could in principle provide an added survival benefit as it has been shown that > 80% of post-operative tumor recurrence is within a 2-cm region surrounding the original GAD-enhancing margin. However, this must be weighed against the risk of potential damage to functional brain tissue.

**Methods:**

In this phase II pilot randomized control trial (RCT), we aim to assess the feasibility of “supra-marginal” resection extending 1 cm beyond the enhancing tumor in adults with radiographic evidence of GAD-enhancing intra-axial tumor consistent with high-grade glioma in a safe anatomical location and a Karnofsky Performance Score > 60. With six academic institutions with established neurosurgical oncology practices in participation, we aim to enroll 72 patients over 2 years. Primary outcomes include evaluating the feasibility of performing a large-scale trial with regard to recruitment, allocation, and outcome documentation as well as safety data. Secondary outcomes include determining if there is an increased survival benefit with supra-marginal resection and impact on quality of life (Modified Rankin Scale (mRS), EuroQol-5D (ED-5D), 30-day all-cause mortality).

**Discussion:**

Recent studies have revealed survival advantages comparing supra-marginal resection to standard attempt at gross total resection (GTR) with no additional perioperative surgical risk; however, the current quality of evidence is low and under-powered. Therefore, there are no current practice guidelines, and the philosophy of surgical resection is guided by individual surgeon preferences on an individual patient basis. This creates additional uncertainty and is potentially detrimental to our patients. This clinical equipoise supports the need for an adequately powered RCT to determine whether a supra-marginal resection can have a positive impact on survival for patients with HGGs. Our pilot RCT will test the feasibility of comparing the standard gross total resection of GAD-enhancing tumors and supra-marginal resection to prepare for a larger definitive multicenter RCT.

**Trial registration:**

ClinicalTrials.gov, NCT04737577. Registered on February 4, 2021

**Supplementary Information:**

The online version contains supplementary material available at 10.1186/s40814-022-01104-1.

## Introduction

High-grade gliomas (HGGs, WHO grade III–IV gliomas) are the most common primary malignant brain tumor in adults [1, 2]. The median survival is only 13–16 months, despite the current standard of maximal safe surgical resection followed by a combination of fractionated radiotherapy and chemotherapy [3, 4]. GTR defined as resection of the GAD contrast-enhancing regions, is an established prognostic marker of overall survival and part of the current standard of care [5, 6]. A meta-analysis of 37 studies (41,117 patients) demonstrated higher 1-year overall survival following GTR compared with subtotal resection (RR 0.62, 95%CI, 0.56–0.69; *P* < .001) [7]. A personalized survival modeling study based on 830 patients demonstrated that *any incremental increase* in the extent of tumor resection improved overall survival [8]. A post hoc analysis of the landmark trial establishing the current standard of care showed that patients in whom GTR was possible gained an additional 4.1 months of survival with adjuvant temozolomide treatment, whereas those with a subtotal resection only gained an additional 1.8 months with this therapy [9]. Maximizing resection of GAD-enhancing regions directly impacts survival while improving the efficacy of modern adjuvant treatment as well [10].

The highly infiltrative nature of HGGs, with histological invasion extending beyond the GAD-enhancing margin on magnetic resonance imaging (MRI), is a known challenge in HGG surgery [11–14]. Therefore, the current definition of GTR based on MRI findings is misleading. It has been shown that > 80% of post-operative tumor recurrence is within a 2-cm region surrounding the original GAD-enhancing margin on MRI [4]. However, there is no consensus on the optimal resection margin beyond the GAD-enhancing region on MRI. Current recommendations regarding the size of the resection margin range from 0.5 to 3.0 cm beyond the GAD-enhancing region [15–17]. Extending the tumor resection limit beyond the GAD-enhancing margin (i.e., supra-marginal resection) could in principle provide an added survival benefit; however, this must be weighed against the risk of potential damage to functional brain tissue. Leveraging the knowledge that certain brain regions are less functional and potentially more amenable to a supra-marginal resection, the adoption of a more aggressive surgical strategy can be conceived in favorable anatomical locations. A meta-analysis based on seven low-quality studies (88 patients total)—none randomized—demonstrated a trend toward improved overall survival favoring a supra-marginal HGG resection, but was limited by failure to measure neurological and functional status in the majority of patients [18].

Clinicians must balance any potential survival benefits of the extent of surgical resection in HGG with the competing risk of potential surgical complications. While a more aggressive philosophy of surgical resection in well-selected patients can potentially improve survival, the potentially added risk of neurological deficit, which could in turn affect the quality of life (QOL) and eligibility for adjuvant therapy, must also be considered. We conducted an independent systematic review of the literature to search for studies investigating whether survival is improved with the extension of surgical resection beyond the enhancing margin of tumor and associated surgical risks. In order to achieve maximal sensitivity, we used a broad search strategy combining terms related to the condition (glioblastoma, high-grade glioma, malignant glioma) with terms describing the extent of surgical resection (marginal, beyond enhancing margin, supra-marginal, MR flair signal margin). Very few studies have examined these risks in detail [18, 19]. Two recent studies evaluated outcomes in a total of 51 patients with supra-marginal resection. One study was a parallel-group prospective study, and the other was a retrospective case series. Both studies revealed survival advantages compared to standard attempts at gross total resection with no additional perioperative surgical risk [20].

The evidence for supra-marginal resection thus far is low-quality and under-powered [18]. Therefore, there are no practice guidelines, and the philosophy of surgical resection is guided by individual surgeon preference on an individual patient basis. This creates additional uncertainty and is potentially detrimental to our patients. In preparation for this pilot study, we have also conducted a survey of Canadian neurosurgeons to evaluate the specialty’s perspective on supra-marginal resections. The most compelling result was that 90% of participants were willing to consider enrolling patients in a randomized controlled trial comparing supra-marginal vs conventional marginal resection (Additional file 6). Together, these findings demonstrate clinical equipoise, supporting the need for an adequately powered RCT to determine whether a supra-marginal resection can have a positive impact on survival for patients with HGGs.

## Objectives

The objective is to conduct a pilot RCT to test the feasibility of comparing the supra-marginal and standard resection approaches in a larger multicenter phase III RCT. The primary feasibility outcome is the number of patients enrolled and successfully allocated to the study interventions per year during the duration of the pilot RCT at each study center.

## Trial design

A randomized (1:1) pilot RCT conducted at six (6) academic institutions with established neurosurgical oncology practices.

## Methods: participants, interventions, and outcomes

### Eligibility criteria

The following are the inclusion criteria:

i) Radiographic evidence of a GAD-enhancing brain tumor consistent with HGG.
ii) Age ≥ 18 to < 80 years.
iii) Karnofsky Performance Score ≥ 60.
iv) The tumor is in an anatomical location that is considered safe for surgical resection (Additional file 1).

The following are the exclusion criteria:

i) Multi-focal tumor, gliomatosis cerebri (≥ 3 lobes of the brain affected), and tumors crossing the midline or with leptomeningeal enhancement
ii) Previous craniotomy for tumor excision (stereotactic biopsy is permitted)
iii) Intraoperative histopathological diagnosis not consistent with HGG
iv) Known metastatic cancer
v) Uncorrectable coagulopathy
vi) Unable to obtain GAD-enhanced brain MRI

**Fig. 1:**
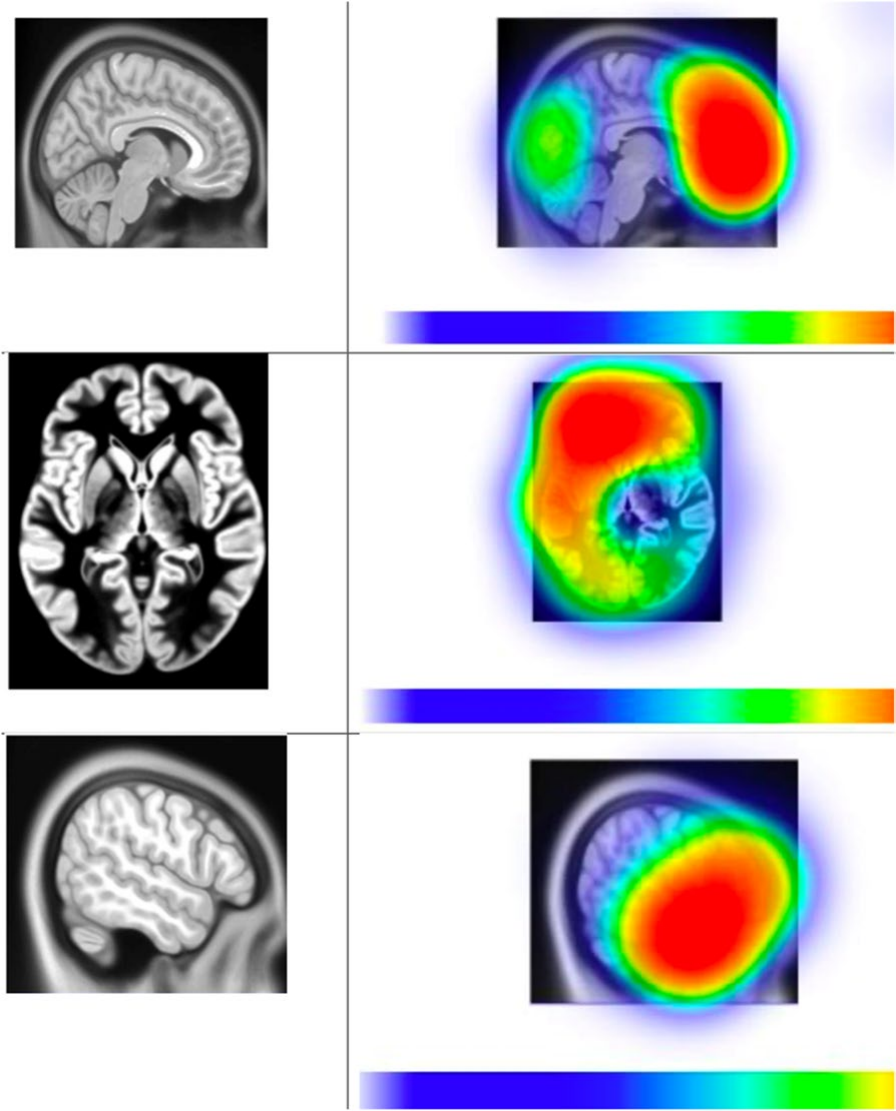
Safe anatomical tumor locations guide. Safe tumor locations include the right and left frontal (excluding supplementary motor area, motor cortex, and Broca’s area), right and left temporal (excluding superior temporal gyrus with the maximal extent of resection posteriorly from the temporal pole of 4–5 cm), and right and left occipital lobes. Increasingly safe regions are indicated in red

The selection of surgeons for this trial will be based on the criteria to demonstrate an adequate level of surgical expertise for the resection of HGGs (Additional file 2). Figure 1 illustrates a guide for safe anatomical tumor locations amenable to standard GTR and supra-marginal resection.

### Recruitment

Research coordinators in each participating center will screen the patients for eligibility in neurosurgical clinics or in the neurosurgical hospital ward on a daily basis for eligibility. Each eligible participant will have the option of connecting support group(s) to allow mutually satisfied discussion and a more comfortable and informed decision to participate. The reasoning for the non-enrollment of eligible patients (either patient or physician related) will be recorded at each center.

### Ethics approval and consent to participate

This study is conducted in accordance with the principles set forth in the Belmont Report: Ethical Principles and Guidelines for the Protection of Human Participants of Research and codified in the Tri-Council Policy Statement and/or the ICH E6.

The study protocol, informed consent form(s), recruitment materials, and all participant materials have been approved by the Ontario Cancer Research Ethics Board (CTO Project ID: 3297).

Prior to involvement in any study-related activities, consent will be obtained in writing for each participant using the current Research Ethics Board (REB)-approved informed consent form. Participants will be given the opportunity to discuss the study with their surrogates or think about it prior to agreeing to participate. They may withdraw consent at any time throughout the course of the study.

### Interventions

#### Explanation for the choice of comparators

This pragmatic RCT is comprised of 2 arms:

1. Supra-marginal resection (intervention arm): planned resection extending beyond the ≥ 95% periphery of the GAD-enhancing region to either at least 1 cm into non-enhancing tissue or to the nearest non-enhancing sulcal boundary/ventricle wall, if these structures are closer than 1 cm
2. Marginal (i.e., GTR) resection: planned resection extending only to ≥ 95% of the GAD-enhancing regions of the tumor without expanding the resection beyond this margin.

#### Intervention description

##### Rationale for supra-marginal definition

More than 80% of HGG tumors recur within a 1–2-cm margin of the original GAD-enhancing regions, and histopathological infiltration can exceed this GAD-enhancing margin by 6–14 mm [5]. A minimum 1-cm margin would balance maximal tumor resection with safety. Given the pattern of tumor cell invasion typically follows white matter tracts, resection beyond the margin of GAD enhancement up to a sulcal boundary or up to the ventricle wall will be accepted for the intervention arm, even if < 1 cm.

#### Criteria for discontinuing or modifying allocated interventions

At their own discretion, participants may withdraw from the study at any time and for any reason. Study participants may also be withdrawn from the study at the discretion of an investigator for reasons such as, but not limited to safety, participant compliance or behavioral concerns. All participants need to have a confirmed final pathological diagnosis of high-grade glial tumor (i.e., grade 3 or higher) to be included in the primary intention-to-treat (ITT) analysis. Participants with any other final diagnosis will not remain in the study, but data will be collected to provide additional safety information.

#### Strategies to improve adherence to interventions

In order to increase the relevance of the trial design to patients, and to increase the likelihood of patient participation, we have identified a patient volunteer who has agreed to meet quarterly to oversee all aspects of research conduct and manage any challenges. We anticipate involvement of a patient stakeholder will specifically enhance the framing of the question, approach to patients and family, nuances in adherence to protocol, and providing support from the beginning to the end for patients.

#### Relevant concomitant care permitted or prohibited during the trial

Use and dose of antiepileptic medications and dexamethasone will be collected. There are no restrictions on concomitant medications, unless they result in preclusion from surgery (e.g., anti-coagulants). Prior chemotherapy or radiation for a brain lesion is not permitted.

### Outcomes

#### Primary feasibility outcome

The primary feasibility outcome is the number of patients enrolled and successfully allocated to the study interventions per year during the duration of the pilot RCT at each study center. This number will determine whether a larger phase III RCT is warranted and feasible.

#### Secondary outcomes

- *Feasibility:* (1) Among all eligible patients, the proportion of patients (i) consenting to participate and (ii) completing all scheduled follow-up assessments. Reasons for non-enrollment of all eligible patients will be recorded (e.g., physician refusal, patient’s preference, inability to meet study timelines). (2) The proportion of patients in each group receiving the assigned extent of surgical resection according to the randomized allocation, as assessed by the volume of residual contrast enhancement in 1st post-operative MRI and compared to the preoperative MRI. Due to the pragmatic nature of this trial, we use intention to treat as a measure of supramaximal resection intent. We expect that as a group, these patients should have less enhancing residual volume in post-operative imaging. Reasons for failure to receive the assigned extent of surgical resection will be recorded (e.g., physician refusal, technical challenges).
- Preliminary *efficacy:* Assessment of the (1) overall survival and (2) progression-free survival based on the Modified Response Assessment in Neuro-oncology (mRANO) criteria [21]. The date of death will be obtained from hospital records, outpatient follow-up clinic notes, or provincial cancer registries. Progression-free survival will be measured based on the mRANO criteria [21] during regular interval clinical and MRI follow-up (according to each institution’s established practice) until death or 12 months after surgery.
- *Safety:* (1) National Institute of Health Stroke Scale (NIHSS), (2) mRS, (3) EQ-5D, and (4) 30-day all-cause mortality. Utilizing three independent scales for the assessment of morbidity will ensure that we capture any objective neurological deficits along with their impact on function and quality of life. It will be critical to ensure that a supra-marginal resection does not lead to an increased 30-day mortality rate, which is a time frame that is most likely to be related to adverse surgical events.

Specifically, we will measure the changes at each assessment relative to preoperative baseline values in the following scales:

- The NIHSS at days 2 ± 1 and 30 ± 5 after surgery (conducted during in-person follow-up). The NIHSS is a validated tool for measuring neurological outcomes that was originally developed for rapid grading of stroke symptoms but has since been adopted in surgical clinical trials [9].
- The mRS (assessed either in-person or over the phone) at 6 and 12 months after surgery. The mRS provides a measure of global disability and has been widely used to assess the outcomes after stroke and other neurological problems. The scale consists of six grades from 0 (no symptoms) to 5 (severe disability); 6 indicates death [22].
- The EQ-5D (assessed either in-person or over the phone) at 6 and 12 months after surgery. The EQ-5D is a generic instrument used to measure health-related QOL, designed for self-completion by respondents (face-to-face or over telephone interview, also available in proxy version through caregiver). It has five dimensions: mobility, self-care, usual activities, pain or discomfort, and anxiety or depression. This tool rates the impact of disease on a scale of 0 to 1 with a lower score indicating a greater effect on the health.
- Where feasible, we will also attempt neuropsychological assessments pre- and post-operatively to detect the subtle differences potentially attributable to a supra-marginal resection.

iv) Health economics: The future definitive RCT will also assess the incremental cost-effectiveness ratio at 6 months. This will be analyzed to determine whether a supra-marginal resection has the potential to decrease societal costs in the long term. Cost data will be collected, in addition to the quality of life (EQ-5D) at 6 and 12 months, which would enable the calculation of the incremental cost-utility ratio (ICUR) of the two interventions. We plan to conduct a full-scale health economic analysis in the future large-scale trial, based on both direct (e.g., operating room, nursing, inpatient hospital stay) and indirect costs (e.g., potential loss of income) costs. Utilities will be calculated based on the EQ-5D, and cost-utility will be based on the perspective of the “idealized insurer”/third-party payer (e.g., the provincial government payer). Given the short survival of HGG patients, discounting will be at 1% with provisions for subgroup analyses.

### Participant timeline

Both intervention and control treatments will terminate at the completion of the surgery. Subsequent treatments will be the same for both groups according to usual clinical practice.

The following clinical and radiological endpoints will be the focus of the larger randomized controlled trial, but will also be measured on all patients during this pilot trial:

- *Clinical*: (a) Neurological status (NIHSS) at days 2 and 30 after surgery [23]. (b) Mortality status at 30 days after surgery and every 3 months thereafter until last follow-up after surgery. (c) Disability (mRS) and quality of life (EQ-5D) at 6 and 12 months after surgery [24].
- *Radiological*: GAD-enhanced brain MRI will be obtained within 72 h after surgery (“first post-operative MR”) and at regular intervals according to each institution’s established practice. This is typically performed every 3 months, or sooner if clinically indicated. Additional post-operative MRIs will be conducted according to each institution’s protocol and reviewed centrally. Additional MRIs are permitted, as needed. Progression-free survival is defined as the number of days from the date of randomization to the date of earliest disease progression based on the mRANO criteria [24] or to the date of death due to any cause, if progression does not occur.

### Schedule of events

**Table:** 

Evaluation	14 days prior to surgery/randomization (screening/enrollment)	Day 0: surgery day (randomization)	Days 1–3: post-OP 1^**st**^ assessment	Day 30 (± 5 days) 2^**nd**^ assessment	6 months (± 2 weeks) post-surgery 3^**rd**^ assessment Collect outcomes	12 months (± 1 month) post-surgery	24 months: annually after (until death, completion of the pilot phase, or lost to F/U)
*Eligibility criteria, medical history, demographics*	X						
*Neurological exam (NIHSS)*	X		X	X			
*MR scan*	X^1^			X ^1^	X^1^	X^1^	X^1^
*Concomitant meds (steroid/antiepileptic)*	X			X	X		
*Received randomly assigned treatment*		X					
*Pathology report*				X			
*Surgical report*				X			
Adverse surgical* adjuvant tx** events			X*	X*	X**	X**	X**
*Outcome assessment (clinical secondary outcomes) (EQD-5-mRS)*					X	X	X
*Death*				X	X	X	X
*Progression free survival*				X	X	X	X
*Timing of RTX*				X	X	X	X
*Timing of chemotherapy*				X	X	X	X

*These adverse events only relate to surgery

**These adverse events only relate to adjuvant radiation and/or chemotherapy

^1^These images are obtained through routine clinical practice

**Table:** 

***Radiation therapy***			
***Time***	***6 months***	***12 months***	***24 months***
***Received > 90% of planned dose***			
***Early discontinuation < 50%***			
***Disease progression***			
***Toxicity***			
***Others***

**Table:** 

***Chemotherapy (temozolomide) therapy***			
***Time***	***6 months***	***12 months***	***24 months***
***Received > 90% of planned dose***			
***Early discontinuation < 50%***			
***Disease progression***			
***Toxicity***			
***Others***			

### Safety criteria

During this pilot study, several safety precautions are being implemented:

i) A clearly defined definition of “surgically safe” anatomical criteria.
ii) Confirming anatomical suitability by a second designated neurosurgeon centrally.
iii) A central adjudication committee, comprising two qualified neurosurgeons, will confirm the suitability of all enrolled cases on an annual basis.
iv) Establishing appropriate surgical expertise and volumes for participating surgeons (Additional file 2),
v) Mandating minimum intraoperative use of 3D neuronavigation and neurophysiologic monitoring to guide resections for all patients.
vi) Incorporating multiple interim safety analyses by the Data and Safety Monitoring Board (DSMB).

### Procedures common to both groups

Imaging, concurrent non-experimental medications, and any adjuvant post-operative care, i.e., external beam radiation therapy plus concomitant and adjuvant temozolomide) including timing, will be according to the standard of care and be recorded. All surgeries will be performed by designated surgeons at each center meeting certain pre-defined surgical skill qualifications (Additional file 2). All surgeries will be performed with the help of intraoperative 3D neuronavigation tools and neurophysiologic monitoring defined by the continuous assessment of the functional integrity of the neural pathway. Other intraoperative adjuncts (e.g., fluorescence-guided, intraoperative MR) are allowed but not required (Additional file 3). The surgical technique (not the extent) for tumor removal is similar in both arms, minimizing any learning curve effect during the trial. Post-operative imaging follow-up with contrast-enhanced brain MRI will be conducted as per institutional practices at regular intervals.

### Sample size

The sample size is based on six sites enrolling patients in this pilot study. All six proposed pilot trial sites are academic tertiary care centers with at least 20–30 malignant glioma surgeries per year (assuming 5 per 100,000/year incidence of new HGG) out of which about one-third are eligible, and assuming that 70% will consent in the 2 years of active recruitment, we will be able to enroll between 45–105 patients. Based on our observational phase I trial (Additional file 4), we were able to enroll at least 1 patient per 2 months, and therefore, a sample size of 72 patients (36 per group) should be feasible for this pilot RCT. If at the end of the study period we recruit 72 patients, we will be 95% confident that these sites will be able to recruit over the same time interval between 56 and 91 patients.

### Assignment of interventions: allocation

#### Sequence generation

Eligible patients will be approached for enrollment either in the preoperative clinic or after hospital admission. Surgery will take place within 14 days of enrollment. Randomization will be done within 3 days prior to the scheduled surgery and will occur in a 1:1 ratio.

#### Concealment mechanism

Randomization will occur up to 3 days prior to surgery to avoid selection bias. Allocation will be concealed from all patients. For sites where randomization is completed by the surgeon, allocation will be concealed as much as possible from the research team. This will be further supported using a template for surgical documentation, ensuring adequate documentation without any reference to treatment allocation (Additional file 5). For sites where randomization is completed by the research team, it will not be possible to conceal allocation from the research team. We will use the ITT principle in analyzing the outcomes and make every effort to obtain secondary outcomes. For all eligible non-enrolled patients, the reason for non-participation by either physicians or patients will be documented to develop strategies to minimize their occurrence in the larger trial. Any systematic differences between baseline characteristics of eligible enrolled and not-enrolled patients will be documented.

### Assignment of interventions: blinding

#### Who will be blinded

Trial participants will be blinded to their intervention assignment. Surgeons cannot be blinded as they are performing the allocated intervention, but allocation will be concealed as much as possible from the research team using templates for documentation without reference to treatment allocation.

#### Procedure for unblinding if needed

Blinding of treatment allocation may only be broken with the permission of the principal investigator if the safety of the participant is at risk and providing a treatment plan requires knowledge of study allocation.

### Data collection and management

#### Plans for assessment, collection of outcomes, and data management

The study case report form (CRF) is the primary data collection instrument for the study. Study personnel, including data entry team members, will use source documents to complete case report forms (CRFs). The information outlined in the schedule of events will be documented in the CRFs.

#### Plans to promote participant retention and complete follow-up

To ensure that the trial can successfully recruit the target sample size, only committed high-volume academic centers have been recruited to participate. The rate of retention in this study will be used to optimize compliance in future larger trials. The outcomes likely most vulnerable to loss to follow-up are anticipated to be the mRS and EQ-5D, which require follow-up at 6 and 12 months. Both of these can be obtained using phone communication, which has similar reliability to in-person assessments. For the assessment of preliminary efficacy (progression-free and overall survival), we will establish contact with the patient or the substitute decision-maker (SDM) on at least 3 occasions, especially if the scheduled MRI appointment is missed. Efforts will be made to facilitate alternative MRI dates or provide locations closer to the patient’s address. If the patient’s status is unknown, study sites will issue certified registered letters to their address and search for the death of a patient in local/regional/national death registries or other public collections of such data as permitted by local privacy laws.

#### Confidentiality

Information about study participants will be kept confidential and managed according to the requirements of the Personal Health Information Protection Act of 2004 (PHIPA) and the Research Ethics Board.

#### Plans for collection, laboratory evaluation, and storage of biological specimens for genetic or molecular analysis in this trial/future use

Plans for collection, laboratory evaluation, and storage of biological specimens for genetic or molecular analysis will be in accordance with standard clinical procedures at each academic hospital site.

## Statistical methods

### Statistical methods for primary and secondary outcomes

#### Primary outcome

Descriptive statistics will be used to report the rate of randomization (patients per month) as overall and per site numbers using medians and interquartile ranges (IQR).

#### Secondary outcomes

Descriptive statistics (with corresponding standard deviations (SD) or IQR) will be used for continuous variables while frequencies and proportions (and 95%CIs) will be used to report categorical variables. A priori subgroup descriptive comparisons will include our randomization strata, along with the impact of duration of adjuvant therapy (e.g., greater or less than 6 cycles of temozolomide chemotherapy). This study will not be powered to make meaningful inferences on the superiority of either intervention. However, to have a better estimate of event rate (death) over time in a larger phase III trial, we will perform time-to-event analyses to evaluate the primary outcome for the future trial of overall survival time. We will interpret the results of each outcome as follows.

#### Adherence to protocol

We will define successful adherence as follows:

i) Documenting every scheduled clinical assessment on day 2, day 30, 6 months, and 1 year (if alive) in at least 90% of patients.
ii) *Implementation of the study protocol* by measuring in at least 90% of enrolled patients the planned primary outcome measure for the larger trial (i.e., overall survival). This rate is comparable to the ability to obtain an outcome in previous large glioma studies [9]

Any reasons for non-adherence to the protocol will be recorded to improve the future larger trial. We will also capture the amount of personnel time required to screen for, consent, enroll patients, complete study procedures, and data collection for the first 5 patients at each site. A strategy to refine the final protocol for the full-scale trial based on the results of the each of above steps will be planned.

The decision to progress to the larger phase III RCT will be based on (1) reaching the primary feasibility target of recruiting at least an average of 1 patient per 2 months per year in each of 6 centers during 2 years of active study participation at each center and (2) no safety or ethical concerns are raised by the Data and Safety Monitoring Board (DSMB) that cannot be rectified. For the future trial, we plan to use overall survival difference (i.e., hazard ratio) to determine the relative efficacy of the interventions and will use data from the pilot study to estimate the required sample size. A meta-analysis assessing 6-month PFS has favored GTR over subtotal resection (STR) (relative risk (RR) 0.72) [7]. For a supra-marginal resection to have a meaningful impact on the survival of HGG patients, a similar improvement of 0.7 hazard ratio would be desired. We calculated that a total of 295 patients (247 deaths) would be required for the trial to have 80% power to detect a hazard ratio of 0.7 (base median overall survival of 14 months) at a two-sided alpha 5% level and planned average length of follow-up of 2 years. Based on our plan to recruit 10 Canadian sites for the phase III study, with an average annual enrollment rate per site of 6 subjects with 2 active surgeons, we anticipate meeting the full enrollment target within 5.5 years. Assuming that there are no major changes to the pilot protocol, we will include the patients’ data in this future larger RCT.

### Interim analyses

To enhance monitoring of safety in the supra-marginal resection group, we plan to submit safety reports every 6 months to the DSMB including 30-day mortality data and all serious adverse events (both expected and unexpected) for their review. The DSMB will have formal annual meetings unless they decide to have them earlier based on the submitted safety reports. The initial DSMB meeting will occur either following accrual of the first 10 participants or 6 months after initiation of the study, whichever comes first.

This study will be stopped prior to its completion if (1) the intervention is associated with adverse events that call into question the safety of the intervention, (2) difficulty in study recruitment or retention will significantly impact the ability to evaluate the study endpoints, (3) any new information becomes available during the trial that necessitates stopping the trial, or (4) other situations occur that might warrant stopping the trial.

### Methods in analysis to handle protocol non-adherence and any statistical methods to handle missing data

All randomized patients will be included in the final analysis according to the ITT principle. Patients who are randomized but who were subsequently determined not to meet the eligibility criteria will not be included in the primary analysis but will be followed to assess safety endpoints.

### Plans to give access to the full protocol, participant-level data, and statistical code

The authors are open to de-identified data including granting public access to the full protocol, participant-level dataset, and statistical code as long as requesting investigators have an IRB-approved protocol.

### Adverse event reporting and harms

Adverse event information will be entered into the CRF in a timely manner and no later than 15 days from the time the investigator becomes aware of the event. Information about any unexpected serious adverse events that are attributed to the study intervention will be entered into the CRF in a timely manner/within 72 h from the time the investigator becomes aware of the event.

### Frequency and plans for auditing trial

Site monitoring will be conducted to ensure the safety of human study participants and the protection of their rights and well-being. Monitoring will verify that collected study data is accurate, complete, and verifiable by source documentation and that the study is conducted in accordance with the protocol and operating procedures. The delegated monitor will evaluate the study processes and documentation based on the approved protocol/amendment(s), the International Council for Harmonisation of Technical Requirements for Pharmaceuticals for Human Use (ICH), E6: Good Clinical Practice guidelines (GCP), and institutional policies.

### Dissemination plans

Plans to communicate the trial results to participants, health care professionals, and the public will be via publications which will adhere to the Uniform Requirements for Manuscripts Submitted to Biomedical Journals of the International Committee of Medical Journal Editors.

## Discussion

Recent studies demonstrate survival advantages comparing standard gross total resection of GAD-enhancing tumor to supra-marginal resection with no additional perioperative surgical risk [20]. However, the current quality of evidence is low and under-powered, limited to prospective studies and retrospective case series. There are no current practice guidelines and the philosophy of surgical resection is guided by individual surgeon preferences on an individual patient basis. This creates additional uncertainty and is potentially detrimental to our patients as supra-marginal resection could potentially increase the risk for surgery-related post-operative deficit or conversely potentially increase overall survival. This clinical equipoise demonstrates the need for an adequately powered RCT to determine whether a supra-marginal resection can have a positive impact on survival for patients with HGGs. Our pilot multi-center RCT aims to test the feasibility, including recruitment potential and safety of comparing standard gross total resection of GAD-enhancing tumor versus supra-marginal resection. The aim is to ultimately prepare for a larger, definitive multicenter RCT.

## Trial status

The trial was registered with ClinicalTrials.gov (NCT04737577) on February 4, 2021.

## Supplementary Information

**Additional file 1.** Safe anatomical locations guide.

**Additional file 2.** Surgeon credentials.

**Additional file 3.** Standard operating procedures.

**Additional file 4.** Confidence intervals.

**Additional file 5.** Surgical documentation template.

**Additional file 6.** SUMIT survey.

## Abbreviations

AE: Adverse event/adverse experience
CCTS: Centre for Clinical Trial Support
CRF: Case report form
DSMB: Data and Safety Monitoring Board
eCRF: Electronic case report form
EQ-5D: EuroQol-5D
GAD: Gadolinium
GCP: Good Clinical Practice
GTR: Gross total resection
HGG: High-grade glioma
ICF: Informed consent form
ICH: International Council for Harmonisation
IQR: Interquartile ranges
ITT: Intent-to-treat
mRANO: Modified Response Assessment in Neuro-oncology
MRI: Magnetic resonance imaging
mRS: Modified Rankin Scale
NIHSS: National Institutes of Health Stroke Scale
pCRF: Paper case report form
PHI: Personal health information
PHIPA: Personal Health Information Protection Act of 2004
PI: Principal investigator
QOL: Quality of life
RCT: Randomized controlled trial
REB: Research Ethics Board
SAE: Serious adverse event/serious adverse experience
SDM: Substitute decision-maker
SI: Site investigator
SMR: Supra-marginal resection
SRI: Sunnybrook Research Institute
STR: Subtotal resection
