# Supplementary material for: A phase II pilot randomized controlled trial to assess the feasibility of the “supra-marginal” surgical resection of malignant glioma (G-SUMIT: Glioma supra marginal incision trial) study protocol
Source: Pilot Feasibility Stud. 2022 Jul 5;8:138. doi: 10.1186/s40814-022-01104-1 (PMC9254510; doi:10.1186/s40814-022-01104-1)
Supplement: Supplementary file 1 — Additional file 1. Safe anatomical locations guide. [file 40814_2022_1104_MOESM1_ESM.pdf]

To establish a consensus, participants were asked to select up to 10 regions in the figures below that would be considered potentially safe for more aggressive tumor removal.

Standard template provided	Regions selected (N=30)
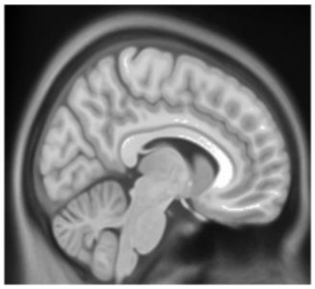	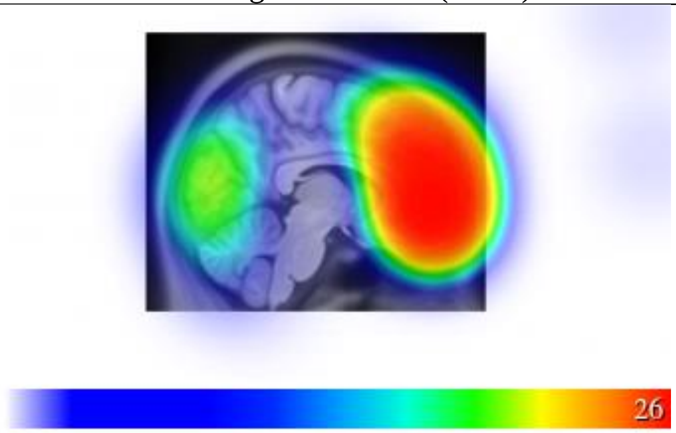
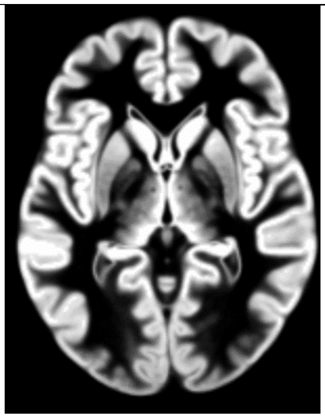	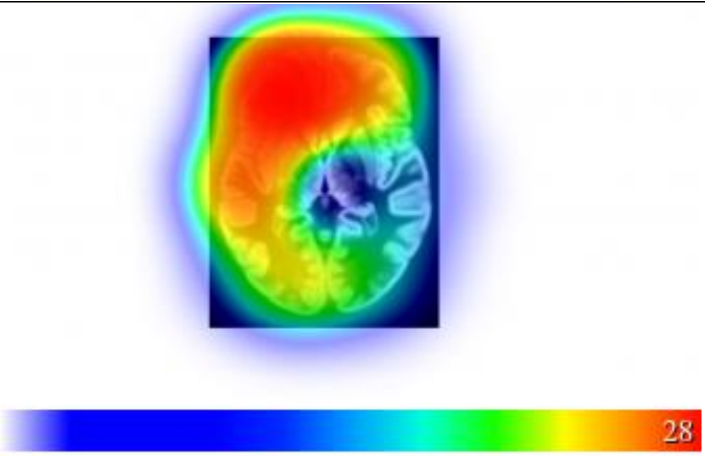
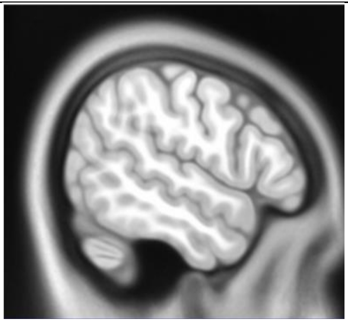	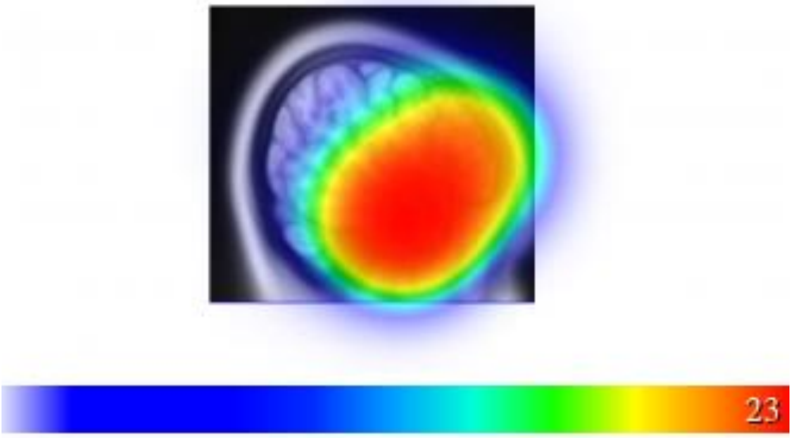

Based on the above results, the following regions have been identified as "safe":

1. Right Frontal lobe
    a. The Supplementary Motor Area will be excluded

2. Right temporal lobe
    a. The superior temporal gyrus will be excluded
    b. Maximal extent of resection posteriorly from the temporal pole will be 4-5cm
3. Right Occipital lobe
4. Left Frontal lobe
    a. The Supplementary Motor Area will be excluded
    b. The Broca's region (Inferior frontal gyrus) will be excluded
5. Left Temporal lobe
    a. The superior temporal gyrus will be excluded
    b. Maximal extent of resection posteriorly from the temporal pole will be 4-5cm

The exclusions above do not apply if the tumor has already invaded these structures and resulted in attributable functional deficit that is deemed irreversible by treating surgeon.
