# Supplementary material for: A phase II pilot randomized controlled trial to assess the feasibility of the “supra-marginal” surgical resection of malignant glioma (G-SUMIT: Glioma supra marginal incision trial) study protocol
Source: Pilot Feasibility Stud. 2022 Jul 5;8:138. doi: 10.1186/s40814-022-01104-1 (PMC9254510; doi:10.1186/s40814-022-01104-1)
Supplement: Supplementary file 2 — Additional file 2. Surgeon credentials. [file 40814_2022_1104_MOESM2_ESM.pdf]

Provisional Certification Status of qualified neurosurgeons

In order for the surgeon to receive provisional certification, he/she must complete the following steps *prior to* performing surgery on a study subject:

1. Confirmation of a at least one year of oncology fellowship with a minimum of 15 glioma surgery in past 2 years or minimum of 5 years in practice and a minimum of 15 surgical procedures within specialty practice annually for prior 2 years.
2. Providing brief discussion of any major surgery-related complications and final outcome (of those complications) in their last 2 years
