# Supplementary material for: A phase II pilot randomized controlled trial to assess the feasibility of the “supra-marginal” surgical resection of malignant glioma (G-SUMIT: Glioma supra marginal incision trial) study protocol
Source: Pilot Feasibility Stud. 2022 Jul 5;8:138. doi: 10.1186/s40814-022-01104-1 (PMC9254510; doi:10.1186/s40814-022-01104-1)
Supplement: Supplementary file 3 — Additional file 3. Standard operating procedures. [file 40814_2022_1104_MOESM3_ESM.pdf]

Standard operating procedures

Preoperative:

All aspects of patient care will be similar. These are outlined below.

Clinical:

- A focused clinical history, physical exam, and neurological exam
- A baseline NIHSS assessment
- Preoperative laboratory assessments (CBC, electrolytes, Creatinine, INR/PTT)
- Baseline chest x-ray and electrocardiogram

Medical:

- Anti-epileptic medications, as needed
- Corticosteroids, as needed

Imaging:

- MRI brain, with and without gadolinium contrast, conducted within 72 hours prior to surgery and randomization, as part of the usual standard of care for intraoperative neuro-navigation.
- At the very least, sequences must include structural sequences (T1- and T2-weighted), fine-cut (1mm slice thickness) T1+Gadolinium sequences, and fluid-attenuation inversion recovery (FLAIR) sequences.

Surgery:

Based on the premise of randomization only upon confirmation of a diagnosis of HGG intraoperatively, the following aspects of surgery should not be affected by patient allocation:

- Patient positioning
- Planned incision pattern and size
- Planned size and location of craniotomy
- Planned use of intraoperative adjuncts such as frameless stereotactic navigation, neurophysiological monitoring, and other adjuncts aimed at increasing the extent of resection

The following general guidelines will be used for surgery:

- After adequate exposure of the tumor, it will be resected using microsurgical technique with the help of 3D frameless stereotactic neuro-navigation to maximize the extent of resection for the GAD-enhancing segment.
- The surgeon may use additional tools, such as intra-operative ultrasound or fluorescence-guided resections, to guide further resection considering that brain shift during debulking can affect the accuracy of the pre-operative neuro-navigation MRIs.
- For patients allocated to the supramarginal arm, an additional circumference of $\geq$1cm will be removed in all quadrants using the aid of the navigation probe AFTER initial gross

total resection (GTR) of the GAD-enhancing segments. Four quadrants of resection cavity will be marked on the navigation screen and a 1 cm virtual mark will be made on all quadrants that will be used a new boundary of additional resection. This virtual marking capacity is available in all 3D navigation systems. This will minimize the effect of brain shift on the extent of planned resection. This technique has been used repeatedly at the lead center and shown to be feasible and reliable based on post operative imaging. Resection to the sulcal limits and the ventricles will be guided by standard anatomical landmarks.

Due to the pragmatic design of this study, surgeons can implement their surgical technique of choice.

Protocol deviation based on safety:

Surgeons can deviate from the allocated intervention during surgery based on the following **safety criteria:** (I) significant neurophysiological changes, (II) cardio-respiratory instability, (III) or intraoperative brain hemorrhage requiring ≥2 units of packed red blood cells.

Postoperative:

All aspects of patient care are to be similar. These are outlined below:

Clinical:

- Daily neurological exam while in hospital
- **Safety**
  - NIHSS assessment at day 2 and 30 after surgery
  - mRS at 6 and 12 months after surgery
  - EQ-5D at 6 and 12 months after surgery
- Laboratory assessments, as needed

Medical:

- Anti-epileptic medications, as needed
- Corticosteroids, as needed

Imaging:

- **Standard immediate postoperative** MRI brain, with and without gadolinium contrast, will be conducted within 72 hours following surgery and randomization
  - At the very least, sequences must include structural sequences (T1- and T2-weighted), fine-cut (1mm slice thickness) T1+Gadolinium sequences, and fluid-attenuation inversion recovery (FLAIR) sequences.
  - A definition of Gross Total Resection is based on evidence of ≥95% resection of the GAD-enhancing 3D volume seen on preoperative imaging
    - The choice to assess volume of GAD-enhancing portions, regardless of allocation to marginal or supramarginal resection, is a practical decision. This is based on the fact that post-resection anatomical shift and postoperative edema, among others, will limit our ability to assess the

extent of supramarginal resection. Furthermore, this is in line with our pragmatic objective of assessing a philosophy of more aggressive resection.

- **6-month PFS** assessment will be based on the mRANO criteria which is comprised of assessing the
  - Percentage change in volume of GAD-enhancing components present at 6 months, compared to the immediate postoperative scan and demonstration of stability of this finding on the follow-up imaging (at least 4 weeks apart)
  - Stability of patient clinical status
  - Stability of corticosteroids dose
- Other imaging modalities, as deemed necessary by the healthcare team, are permitted.

<u>Adjuvant therapy:</u>

Provision of adjuvant therapy will be based on the current standard of care:

- Based on the decision of the oncology team, patients may either undergo combined chemo-radiation or a short course of palliative radiation therapy
- Concurrent chemotherapy and radiation
  - Radiation: 60Gy in 30 fractions (6 weeks)
  - Chemotherapy: Temozolomide 75mg/m^2 once daily, PO (Daily during radiation, followed by 4-week break)
- Maintenance chemotherapy
  - The decision to continue on to maintenance chemotherapy will be contingent on the health status of the patient and the decision of the oncology team
  - Temozolomide 150-200mg/m^2 once daily, PO (on days 1-5 of the 28-day cycle, 6 cycles total, maximum of 12 cycles).
    - 95% is defined as GTR
