# Supplementary figures and images for: A phase II pilot randomized controlled trial to assess the feasibility of the “supra-marginal” surgical resection of malignant glioma (G-SUMIT: Glioma supra marginal incision trial) study protocol
Source: Pilot Feasibility Stud. 2022 Jul 5;8:138. doi: 10.1186/s40814-022-01104-1 (PMC9254510; doi:10.1186/s40814-022-01104-1)

**Confidence interval as a function of Power**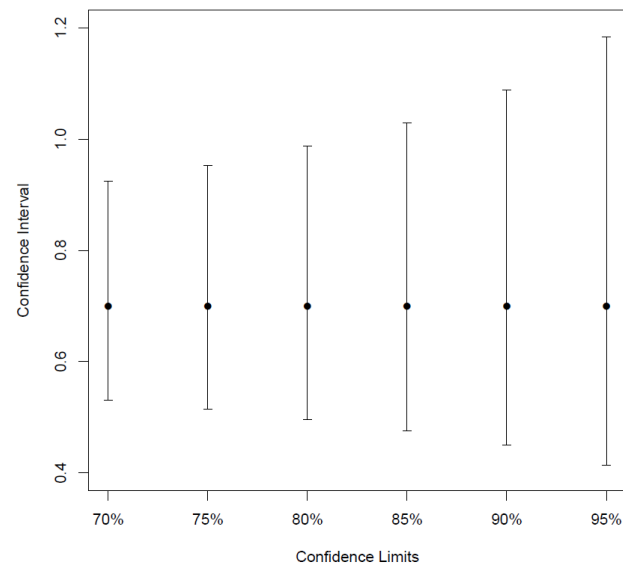

Supplement: Supplementary file 4 — Additional file 4. Confidence intervals. [file 40814_2022_1104_MOESM4_ESM.pdf]
