# Supplementary material for: A phase II pilot randomized controlled trial to assess the feasibility of the “supra-marginal” surgical resection of malignant glioma (G-SUMIT: Glioma supra marginal incision trial) study protocol
Source: Pilot Feasibility Stud. 2022 Jul 5;8:138. doi: 10.1186/s40814-022-01104-1 (PMC9254510; doi:10.1186/s40814-022-01104-1)
Supplement: Supplementary file 5 — Additional file 5. Surgical documentation template. [file 40814_2022_1104_MOESM5_ESM.pdf]

Surgical documentation form

Date:

Surgeon:

Assistants:

Pre-operative diagnosis:

Post-operative diagnosis:

Surgical procedure:

- Please do not describe allocation
- Please limit description of resection to either having achieved a subtotal or gross-total resection

Estimated blood loss:

Intraoperative complications:
