# Supplementary material for: A phase II pilot randomized controlled trial to assess the feasibility of the “supra-marginal” surgical resection of malignant glioma (G-SUMIT: Glioma supra marginal incision trial) study protocol
Source: Pilot Feasibility Stud. 2022 Jul 5;8:138. doi: 10.1186/s40814-022-01104-1 (PMC9254510; doi:10.1186/s40814-022-01104-1)

High Grade Gliomas (HGG, WHO Grade III-IV glioma) is a malignant infiltrative brain tumor that portends a poor prognosis. Accumulating evidence has suggested that any incremental removal of contrast-enhancing portions of tumor can improve the overall survival of patients. However, knowing that tumor cells extend well beyond the contrast-enhancing portion, there have been no robust high-quality systematic studies to determine the impact on survival of removing an additional margin of tissue that is not contrast-enhancing.

Through this survey, we would like to better assess the current philosophy of the neurosurgical community toward more aggressive surgical management of HGG in relatively safe anatomical locations.

The MRI below pertains to a 52-year old male, presenting with headaches, mild confusion, and a left sided pronator drift.

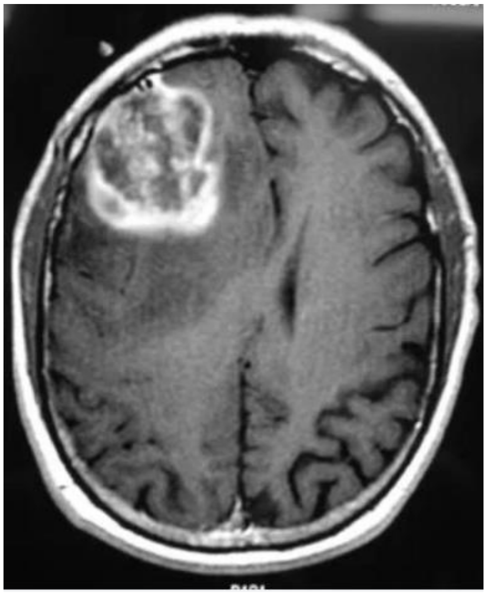

1) On a scale of 1 (Not important at all) to 10 (Very important), how strongly do you feel that complete tumor removal in the above patient is important to survival?

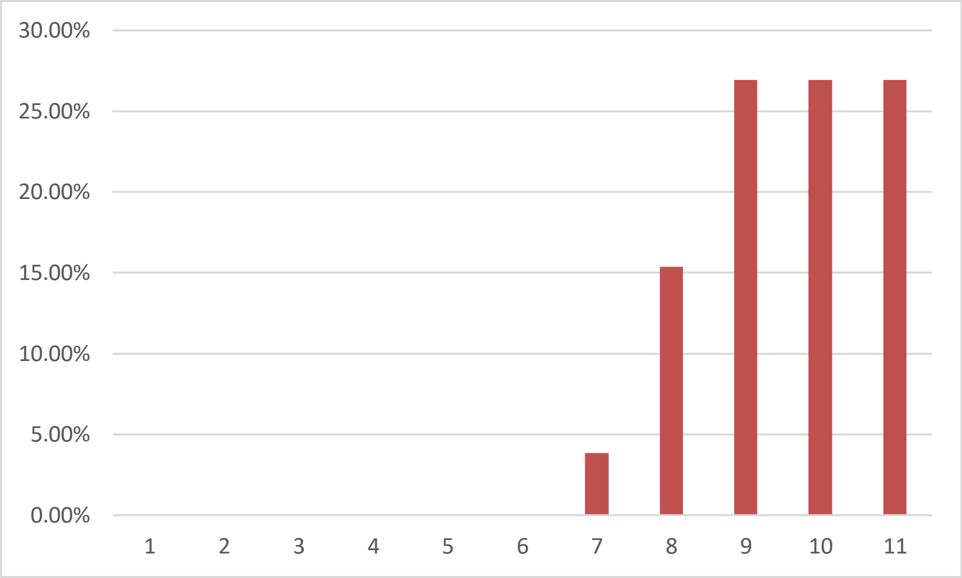

2) In the illustrative case above, how confident are you that you could remove the entire contrast-enhancing portion, without causing new neurological deficits that affect the patient's performance status?

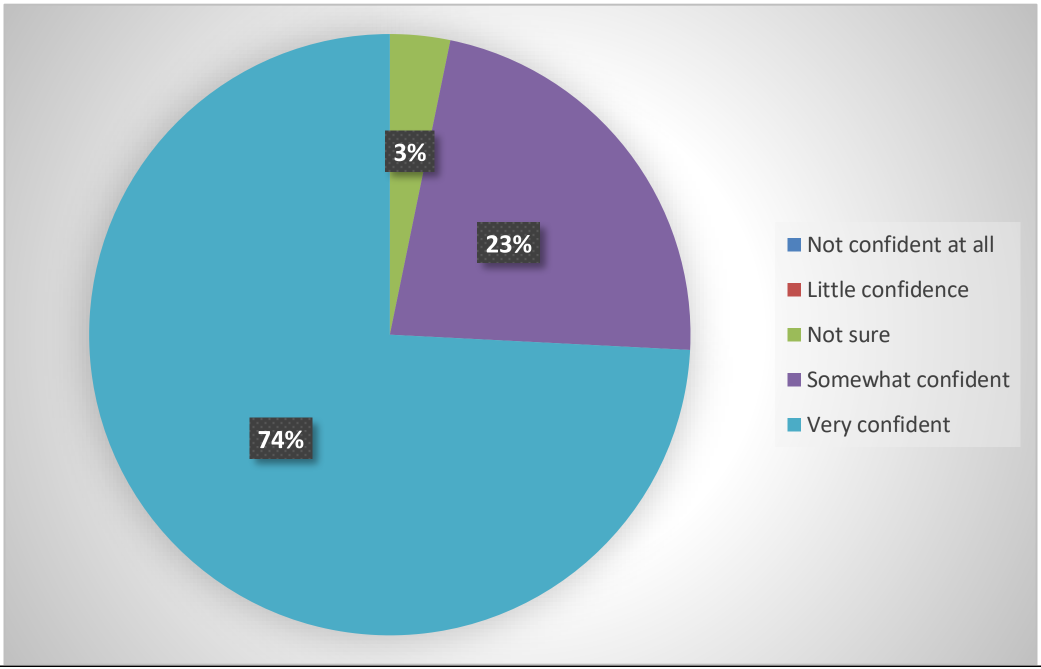

3) In the illustrative case above, how confident are you that you could remove the entire contrast-enhancing portion in addition to a 1 cm margin, without causing new neurological deficits that affect the patient's performance status?

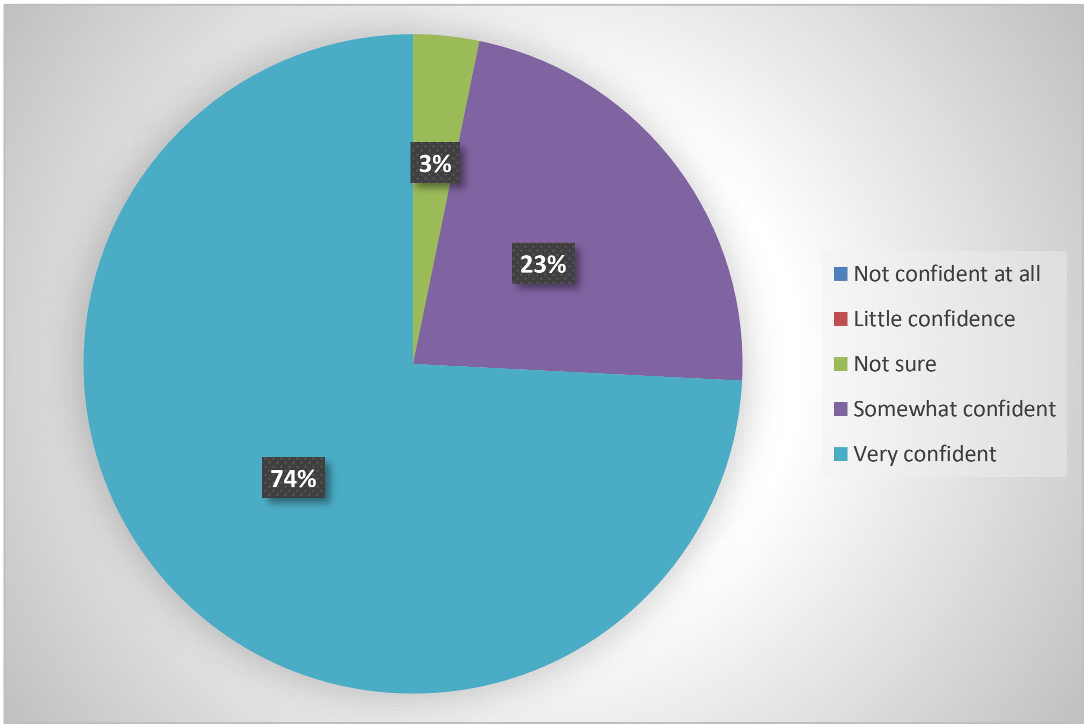

4) Do you believe that currently there is evidence regarding the value of extent of surgical resection of contrast-enhancing tumor in improving the overall survival of patients with HGG?

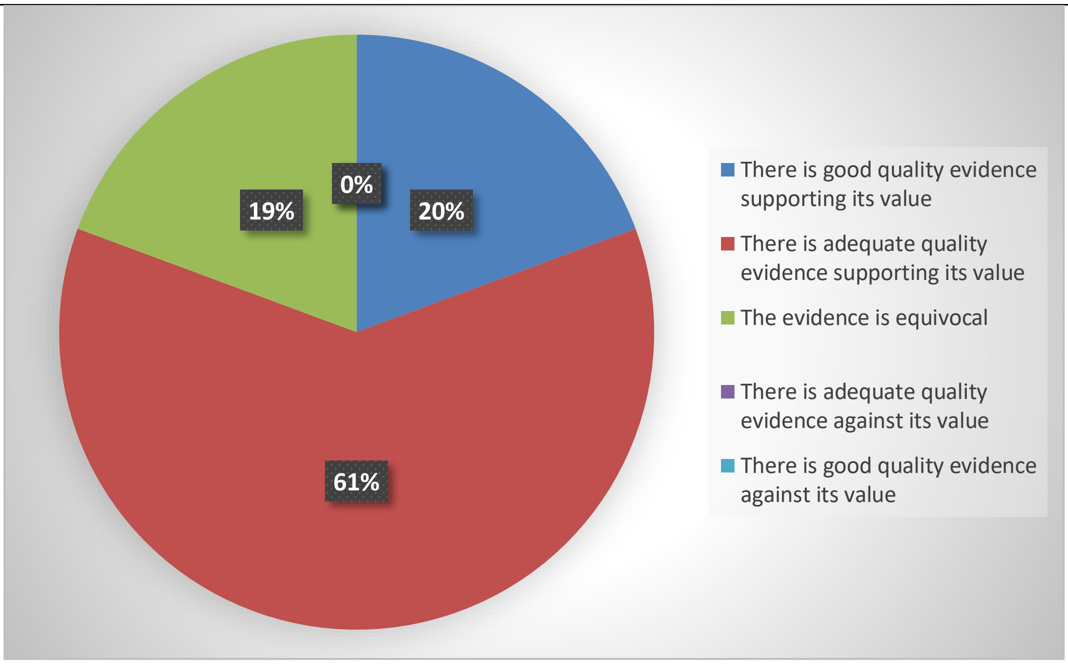

5) How would you grade the EXISTING evidence base supporting the resection of additional tissue, beyond the contrast-enhancing margin of a HGG?

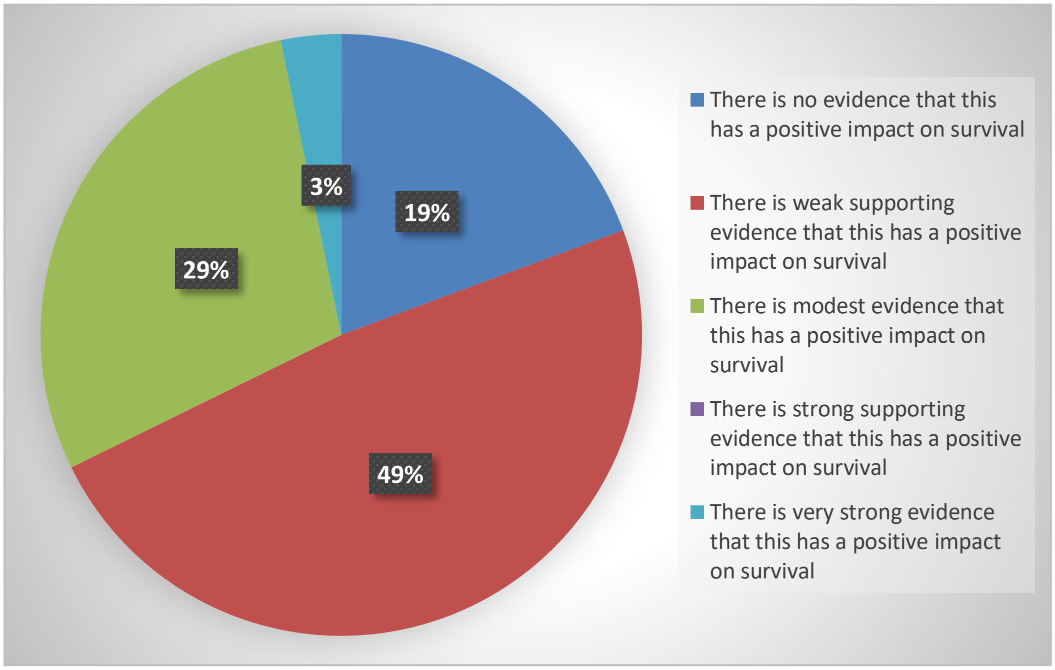

6) How often would you resect tissue beyond the contrast-enhancing portions of the tumor, assuming the tumour resides in a favorable anatomical location?

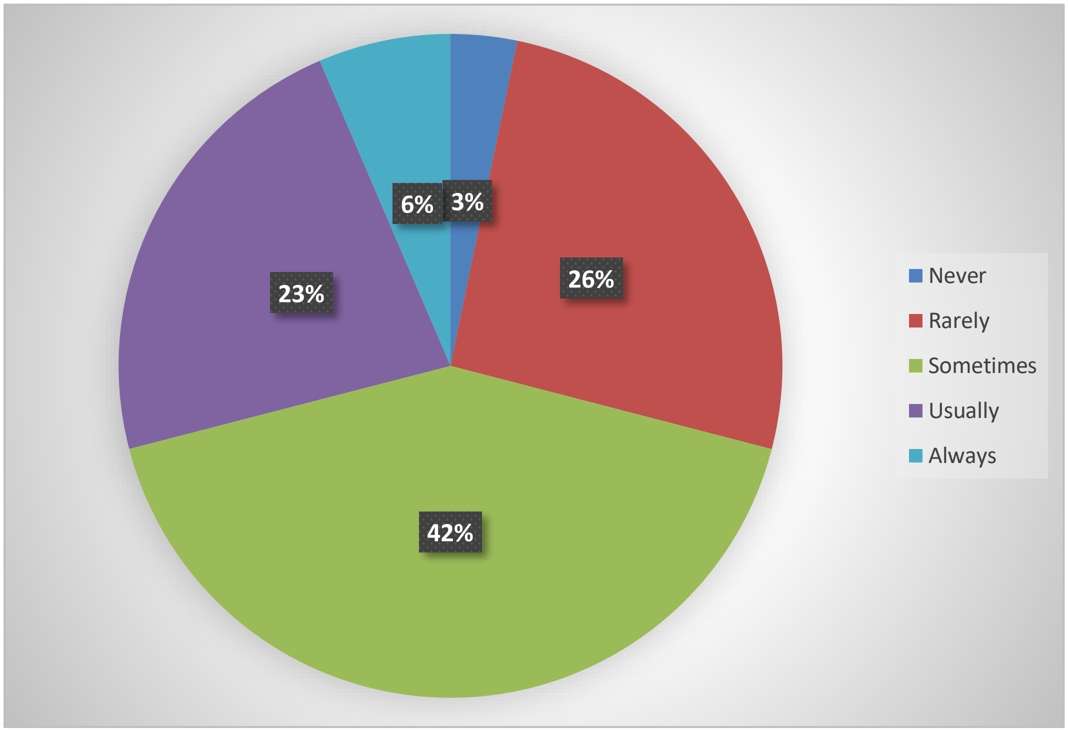

7) Would you consider enrolling patients with tumors residing in safe anatomical locations in a trial to assess the impact of extending surgical resection to beyond the enhancing margins seen on MRI, versus only resecting the contrast-enhancing regions, on survival?

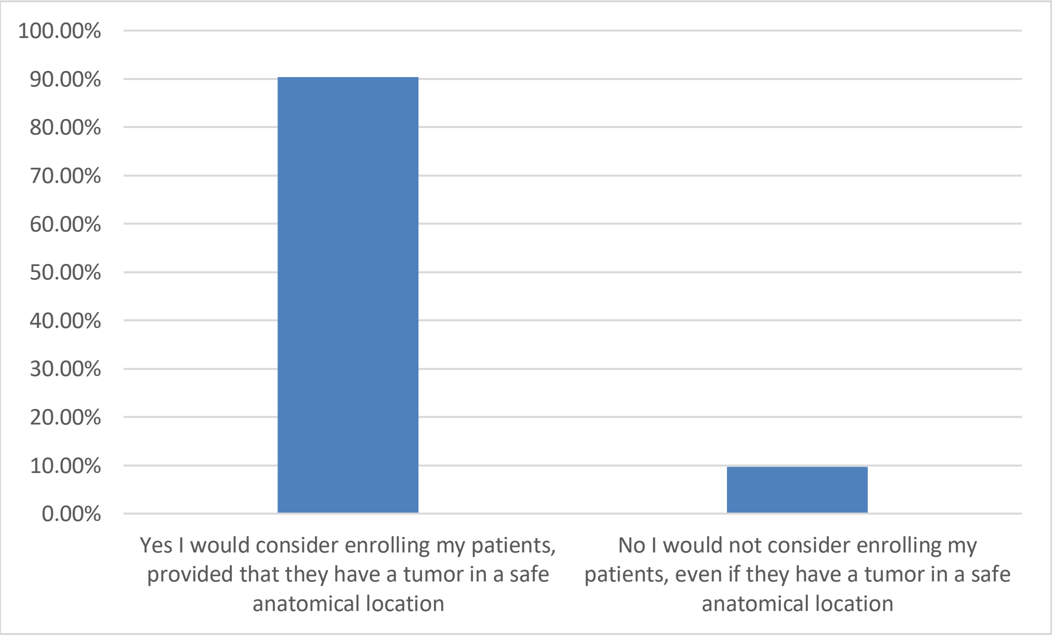

Supplement: Supplementary file 6 — Additional file 6. SUMIT survey. [file 40814_2022_1104_MOESM6_ESM.pdf]
